# Chagas Cardiomyopathy: From Romaña Sign to Heart Failure and Sudden Cardiac Death

**DOI:** 10.3390/pathogens10050505

**Published:** 2021-04-22

**Authors:** Antonia Pino-Marín, Germán José Medina-Rincón, Sebastian Gallo-Bernal, Alejandro Duran-Crane, Álvaro Ignacio Arango Duque, María Juliana Rodríguez, Ramón Medina-Mur, Frida T. Manrique, Julian F. Forero, Hector M. Medina

**Affiliations:** 1 School of Medicine and Health Sciences, Universidad del Rosario, Bogotá 110131, Colombia; germanj.medina@urosario.edu.co (G.J.M.-R.); juans.bernal@urosario.edu.co (S.G.-B.); mjrodriguez@cardioinfantil.org (M.J.R.); jforero@cardioinfantil.org (J.F.F.); hmedina@cardioinfantil.org (H.M.M.); 2 Division of Cardiology, Fundación Cardio-Infantil-Instituto de Cardiología, Bogotá 110131, Colombia; rimedina@cardioinfantil.org (R.M.-M.); fmanrique@cardioinfantil.org (F.T.M.); 3 Internal Medicine Residency Program, Cleveland Clinic Foundation, Cleveland, OH 44195, USA; duranca@ccf.org; 4 Department of Infectious Diseases, Fundación Cardio-Infantil-Instituto de Cardiología, Bogotá 110131, Colombia; aarangod@cardioinfantil.org; 5 Division of Radiology, Fundación Cardio-Infantil-Instituto de Cardiología, Bogotá 110131, Colombia

**Keywords:** Ch-CMP/Chagas cardiomyopathy, Chagas disease, trypanosoma, heart failure, sudden cardiac death

## Abstract

Despite nearly a century of research and accounting for the highest disease burden of any parasitic disease in the Western Hemisphere, Chagas disease (CD) is still a challenging diagnosis, primarily due to its poor recognition outside of Latin America. Although initially considered endemic to Central and South America, globalization, urbanization, and increased migration have spread the disease worldwide in the last few years, making it a significant public health threat. The international medical community’s apparent lack of interest in this disease that was previously thought to be geographically restricted has delayed research on the complex host–parasite relationship that determines myocardial involvement and its differential behavior from other forms of cardiomyopathy, particularly regarding treatment strategies. Multiple cellular and molecular mechanisms that contribute to degenerative, inflammatory, and fibrotic myocardial responses have been identified and warrant further research to expand the therapeutic arsenal and impact the high burden attributed to CD. Altogether, cardiac dysautonomia, microvascular disturbances, parasite-mediated myocardial damage, and chronic immune-mediated injury are responsible for the disease’s clinical manifestations, ranging from asymptomatic disease to severe cardiac and gastrointestinal involvement. It is crucial for healthcare workers to better understand CD transmission and disease dynamics, including its behavior on both its acute and chronic phases, to make adequate and evidence-based decisions regarding the disease. This review aims to summarize the most recent information on the epidemiology, pathogenesis, clinical presentation, diagnosis, screening, and treatment of CD, emphasizing on Chagasic cardiomyopathy’s (Ch-CMP) clinical presentation and pathobiological mechanisms leading to sudden cardiac death.

## 1. Introduction

Chagas disease (CD)—or American trypanosomiasis—accounts for the highest disease burden of any parasitic disease in the Western Hemisphere. Nevertheless, it remains a challenging diagnosis, mostly due to a complex host–parasite interrelationship and low recognition outside of Latin America (LATAM) despite massive migration to developed countries in the last four decades and nearly a century of research.

Although different historical registries indicate that CD was present since the Pre-Columbian era, its first formal description was made by Carlos Chagas in 1908 during an anti-malaria campaign to support a railway track’s construction in the state of Minas Gerais, Brazil [1]. He identified many large hematophagous insects called triatomines that bit individuals (particularly on the face) and later found multiple trypanosomes in their hindgut. He named it *Trypanosoma cruzi* in honor of his mentor Oswaldo Cruz. Later, he made the first formal clinical description of the acute phase and linked the infection with the onset of chronic manifestations [1,2,3]. He became a remarkable doctor and researcher as he had discovered a new infectious disease and described its pathogen, vector, host, clinical manifestations, and epidemiology.

**Figure 1:**
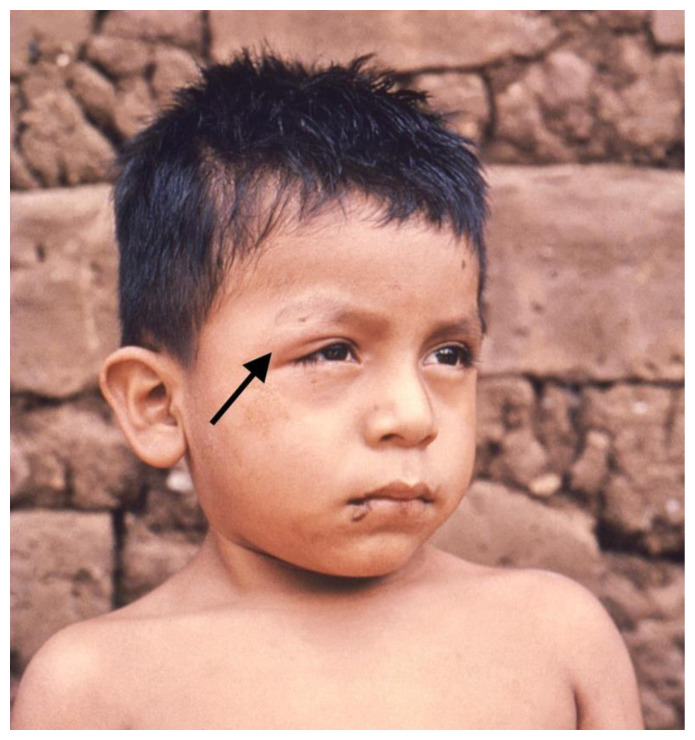
Romaña sign. CDC/Dr. Mae Melvin Image - PHIL. https://phil.cdc.gov/Details.aspx?pid=15814 (accessed on 20 April 2021) https://www.cdc.gov/parasites/chagas/gen_info/vectors/index.html#list (accessed on 16 February 2021).

The acute phase of the infection is typically asymptomatic, and approximately 5% of patients experience mild symptoms, including fever, malaise, and the characteristic unilateral edema of the eyelids that occurs when the insect bites near the eye, also known as the Romaña sign (Figure 1) [4]. Afterward, the chronic asymptomatic infection begins, and about 50% of patients will remain in this phase, characterized by the absence of any clinical signs [5]. Among the long-term manifestations in the chronic phase, Ch-CMP is arguably the most severe form of the disease. It is a condition with a wide range of clinical manifestations, including heart failure, arrhythmias, high degree heart block, thromboembolism due to ventricular aneurysms, and sudden cardiac death (SCD) [6,7].

On average, 25% of chronically infected individuals develop Ch-CMP, making it the leading cause of non-ischemic cardiomyopathy in LATAM [5,8]. The disease is usually restricted to rural and peri-urban tropical regions, closely related to low-income neighborhoods. However, recent globalization, urbanization, and increased migration have spread the disease to other unusual areas such as North America, Europe, Australia, and Japan, forcing healthcare workers in these locations to become more aware of this condition. This review aims to summarize the most recent information on the epidemiology, pathogenesis, clinical presentation, diagnosis, screening, and treatment of CD, emphasizing Ch-CMP clinical presentation and the mechanisms leading to SCD.

## 2. Epidemiology

Chagas disease is part of the list of neglected tropical diseases issued yearly by the World Health Organization (WHO) because of its prevalence in populations with low socioeconomic status, that live in tropical and subtropical regions, with precarious sanitary conditions and are in close contact with infectious vectors [9,10]. Moreover, it is a cause of substantial morbidity and mortality with a significant economic impact on developing countries. Besides, most people at high risk of contagion usually experience multiple barriers to appropriate evaluation, diagnosis, and treatment due to limited healthcare access.

According to the estimates of the 2010 WHO epidemiological update on CD in LATAM, more than five million people infected with *T. cruzi* in 21 Latin-American countries. Argentina, Brazil, and Mexico were the countries with the highest prevalence, followed by Bolivia and Colombia (Table 1) [8,9]. Approximately 20 to 25% of those infected with CD are estimated to have Ch-CMP, which accounts for nearly two million people [8].

These numbers are responsible for a considerable burden on the health system of the affected countries. For instance, in Colombia during 2008, the medical care cost for all CD patients was estimated to be USD 267 million. Additionally, the total cost of prevention programs based on vector control, house improvements, and blood transfusion screening was USD five million [15]. Of note, these programs seem to have had a positive impact on reducing and interrupting transmission, as noticed by the consequent decrease in CD prevalence over the past decades [8,15].

This condition was restricted to specific locations in Central and South America. Its occurrence outside these regions used to be rare and infected patients were easy to identify and trace. However, globalization has expanded the disease to other parts of the world. More than 400,000 CD patients reside outside LATAM, mostly in North America [16]. Although local vector-borne transmission is documented in the southern United States, most infected patients are immigrants from endemic countries or people who acquired the infection through blood transfusion, organ transplantation, or vertical transmission due to maternal infection [13,16]. Despite its increasing prevalence, CD outside of LATAM remains poorly recognized.

The disease itself has no gender predominance, and the most commonly described risk factors for infection are advanced age, rural residency, and a lower education level [6,8,17]. Vector-borne transmission is the most common route for infection, followed by the other previously mentioned mechanisms. The oral transmission is increasingly documented in the last years in different regions in LATAM, probably due to contamination of food and beverages with triatomine feces containing trypanosomes, which subsequently infect the oral mucosa [6,18]. The transmission has importance over the others regarding public health because of feasibility in almost any region in the Amazon, Caribbean, and Andean regions and its relationship with a higher inoculum and more severe manifestations. The severe acute CD secondary to oral outbreaks is associated with higher rates of acute myocarditis, meningoencephalitis, and fatality cases, probably due to the sustained production of inducible pro-inflammatory cytokines, subsequently continued inflammation, and higher parasitization of cardiac muscle and the brain [19]. The largest oral outbreaks in Venezuela in different schools, with approximately 100 people affected, are mostly in children [20,21]. In both cases, epidemiological investigations incriminated contaminated fresh guava juice as the sole source of infection. These reports show that cultural customs such as the preparation and consumption of artisanal food preparations and liquids can lead to substantial illnesses that will impact the community for years, probably leaving higher rates of chronic cardiac morbidity and a higher possibility of congenital transmission in the upcoming years [21].

## 3. Pathophysiology of Infection

### 3.1. Microbiology of Infection

CD is a zoonotic infection caused by *Trypanosoma cruzi*, a protozoan parasite. Other species among this genus that cause disease in humans are *T. brucei gambiense* and *T. brucei rhodesiense,* the etiologic agents of African trypanosomiasis (African sleeping sickness) [22,23]. Its significant genetic variability characterizes *T. cruzi*. The parasite’s natural populations are classified into seven different lineages or discrete typing units (TcI-TcVI and TcBat); lines differ from each other by their geographic distribution, host specificity, and pathogenicity [22,24,25]. TcI is the most abundant and widely distributed in America and is the principal cause of CD in Central America and the northern countries in South America. Simultaneously, TcII, TcV, and TcVI are more prevalent in southern South America [24,26,27].

The clinical implications of this genetic diversity have not been established. Nevertheless, the strains’ heterogeneity is considered one of the main factors involved in the wide range of CD clinical manifestations [25]. For example, it may be responsible for the higher frequency of gastrointestinal disease in LATAM’s southern countries [26,28]. On the other hand, Ch-CMP occurs throughout all the subspecies of *T. cruzi* [28,29]. In this way, the clinical course of chronic infection seems to be the result of the complex interactions between the different *T. cruzi* strains, the host’s immunogenetics, and the eco-epidemiological characteristics of the disorder.

**Figure 2:**
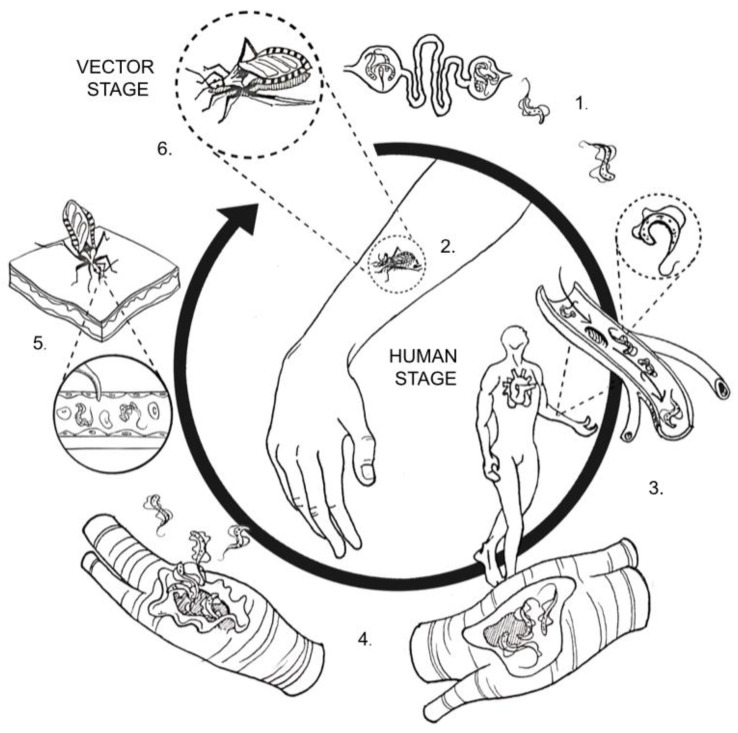
Life cycle of *Trypanosoma cruzi.* 1. In the triatomine’s midgut, trypomastigotes differentiate into epimastigotes, the main invertebrate replicating form, which multiplies by binary fission. Epimastigotes then migrate into the vector’s hindgut, differentiating into trypomastigotes in the vector’s feces. 2. Trypomastigotes excreted in feces enter the host through bite wounds or mucosal surfaces such as the conjunctiva. 3. Trypomastigotes enter the circulation and infect many types of nucleated cells. 4. Inside nucleated cells, trypomastigotes transform into amastigotes and multiply by binary fission, then once again convert to trypomastigotes and cause cell rupture. Trypomastigotes are released into the host’s circulation and can infect other cells to begin a new replicating cycle. 5. When a triatomine vector ingests a blood meal from an infected mammalian host, it becomes infected, completing the parasite’s cycle. 6. Infected triatomine vectors then host *Trypanosoma cruzi*, which proceeds to replicate inside the vector’s gut.

Besides humans, several mammals serve as reservoirs for *T. cruzi,* including armadillos, raccoons, woodrats, some species of rodents, and domestic dogs. Common triatomine vector species belong to the genera *Triatoma*, *Rhodnius*, and *Panstrongylus*, often called kissing bugs [22]. Vector-borne transmission via these infected triatomine bugs is the primary transmission route (Figure 2). These arthropods become infected by sucking blood from animals or humans who have circulating parasites or trypomastigotes. Ingested organisms multiply in the triatomine’s gut and differentiate into an intermediary form (epimastigotes) and then into the infective form of trypomastigotes.

Trypomastigotes are within the feces near the bite wound or intact mucosal tissues such as the conjunctiva. Inside the host, trypomastigotes invade the cells near the inoculation site where they differentiate into intracellular amastigotes that multiply and differentiate into trypomastigotes without the ability to replicate. Finally, these forms are released into the circulation and may infect cells from various tissues and transform into intracellular amastigotes [30,31].

### 3.2. Pathogenesis

The complex host–parasite relationship that characterizes CD determines the tissue injury and, therefore, the myocardial involvement. Local changes include the development of interstitial edema, lymphocytic infiltration, and reactive hyperplasia of lymph nodes [32]. After dissemination, the main sites of tissular involvement are muscle and ganglion cells, including the myocardium, with the characteristic pseudocysts appearance or aggregates of multiplying intracellular parasites [33].

Myocardial inflammation develops over time and involves multiple cellular and molecular mechanisms that contribute to degenerative, inflammatory, and fibrotic responses. Four main pathogenic mechanisms have been proposed to explain Ch-CMP development: cardiac dysautonomia; microvascular disturbances; parasite-dependent myocardial damage; chronic immune-mediated myocardial injury [34].

Altogether, the above mechanisms determine the infection’s clinical manifestations, ranging from asymptomatic disease to severe cardiac and gastrointestinal involvement. Although all of them are relevant in developing the disease, ongoing systemic infection with documented autoimmune reactions plays an outstanding role in the pathogenesis of CD [34,35].

#### 3.2.1. Dysautonomia

Regarding dysautonomia, ganglionic damage and reduction in the subepicardial intramural neuronal count have been documented. Direct parasite damage causes periganglionar and degenerative abnormalities in Schwann cells and nerve fibers due to an antineuronal autoimmune reaction, which may explain the morphological findings [34]. Neuronal loss, which is the principal mechanism of cardiac autonomic dysregulation, occurs predominantly during the acute phase of the infection [32,34,36].

Since the intramural cardiac ganglia are mostly parasympathetic, their damage causes a long-lasting autonomic imbalance that leads to catecholamine-induced cardiomyopathy with impairment of the parasympathetic inhibitory action generally exerted on the sinus node [34]. This phenomenon causes patients to lack the vagal-mediated negative chronotropic response, which usually occurs after transient changes in blood pressure or venous return [6,34]. Furthermore, early parasympathetic impairment could be a mechanism that triggers SCD because of an augmented vulnerability to malignant ventricular arrhythmias [32,34,36,37]. Moreover, it aggravates contraction disturbances and leads to global bi-ventricular systolic dysfunction because of the loss of the homeometric mechanism to physiological stimuli. As a result, the normal physiological response is replaced by more stressful adaptations consisting of heteromeric adjustments that require variations in ventricular volume and shape, potentially leading to chamber dilation, hypertrophy, or both [34].

#### 3.2.2. Microvascular Disturbances

Microvascular disturbances are caused by perivascular inflammation, cell necrosis, and subsequent intimal proliferation and fibrosis, leading to abnormalities in the mechanisms of vasodilation and vasoconstriction and transient microvascular ischemic disturbances of low intensity and short duration [6,34]. These microinfarctions have been postulated to be pivotal in developing ventricular aneurysms because of the unopposed sympathetic overstimulation, a theory that links the microcirculatory and neurogenic hypotheses [34,38].

Among other microcirculatory disorders, occlusive platelet thrombi formation in small epicardial and intramural coronary arteries is typical [32,34]. Increased endothelin production, which mediates arteriolar spasm and inhibits cAMP, with consequent stimulation of platelet adhesion to the vascular wall, has been postulated as the primary pathophysiological mechanism [34]. The absence of obstructive disease at the epicardial level supports the concept of abnormal myocardial flow regulation at the microvascular level [34,39]. It is reasonable to conclude that chronic myocardial hypoperfusion contributes to the characteristic regional left ventricular (LV) dysfunction [34,40].

**Figure 3:**
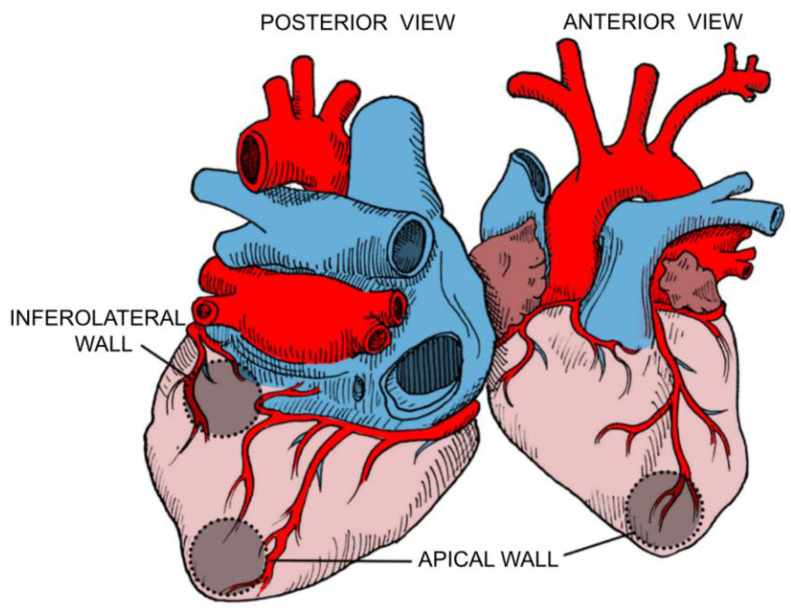
Commonly affected segments and terminal circulation segments.

**Figure 4:**
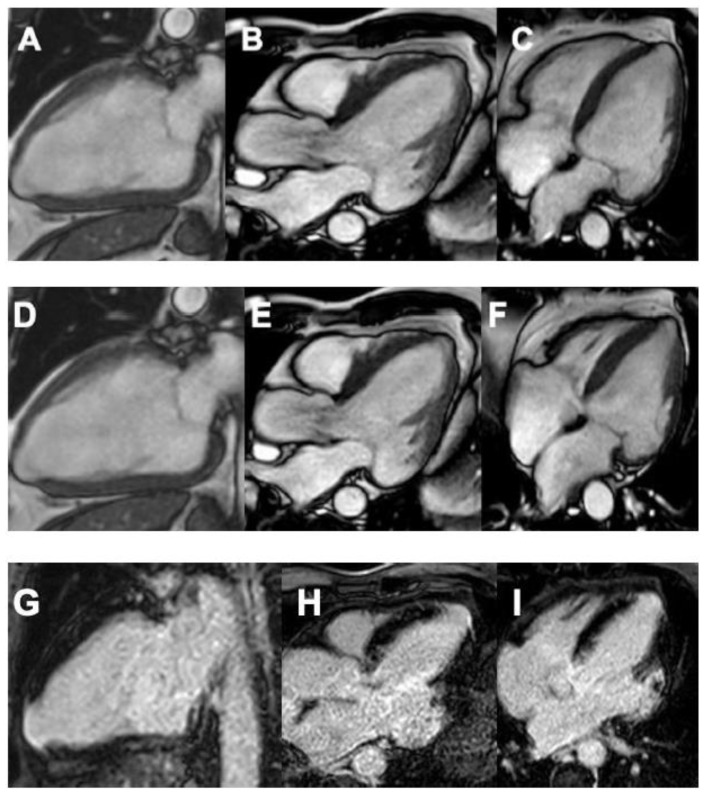
Apical and basal inferolateral aneurysm with the corresponding scar at the same location. (**A**–**C**): CMR cine end-diastolic frame in apical 2, 3, and 4 chamber views, (**D**–**F**): CMR cine end-systolic frame in apical 2, 3, and 4 chamber views, (**G**–**I**): CMR-LGE in apical 2, 3, and 4 chamber views.

Preferable sites for focal fibrosis have been identified in anatomopathological and imaging studies; among all AHA 17 segments, the LV apex and the basal inferolateral wall are the most commonly affected territories. These regions are terminal circulation segments (the apex between the anterior descending and the right coronary artery and the basal inferolateral segment between the right coronary and circumflex artery) (Figure 3). The mechanisms of chronic myocardial inflammation with release of pro-inflammatory cytokines and other mediators resulting from *T. cruzi* infection may cause several episodes of intense microcirculatory vasodilation leading to decreased myocardial blood flow (also known as the “steal” phenomenon) in the distal portions of coronary microcirculation, causing ischemia and fibrosis in these segments [41] (Figure 4).

#### 3.2.3. Parasite Dependent Myocardial Damage

Regarding parasite-dependent myocardial damage, tissue damage and clinical expression of the disease in the acute phase have been related to the degree of parasitemia and tissue tropism, dependent on the parasite and host’s genetic characteristics. Carbohydrate residues in membrane glycoconjugates such as galactosyl, mannosyl, and sialyl in cardiomyocytes participate in parasite entry. The parasite directly alters these surface glycoconjugates, restricting Gal-1 and its inhibitory effect on *T. cruzi* infection over cardiomyocytes. Intracellularly, it takes control of the host cell with the production of cytokines and other molecules, which perpetuate the inflammatory response [34,42]. However, the exact mechanism by which the parasite causes tissue damage in the chronic phase is unclear, and it seems to be not so relevant compared to the chronic immune responses for the development of clinical manifestations in the advanced stages of heart failure [32,34].

#### 3.2.4. Chronic Immune-Mediated Myocardial Injury

Finally, as for the immune-mediated mechanisms, a delayed type-IV hypersensitivity reaction with diffuse mononuclear myocarditis and myocytolysis is the chronic Ch-CMP hallmark. A maladaptive myocardial fibrosing reaction in this chronic inflammatory response is also crucial for understanding the CD involvement. Along with the evidence of immunoglobulin and complement deposition in myocardial tissue, these findings constitute evidence for the role of immunologic mechanisms in the Ch-CMP’s pathogenesis [34].

It is well known that the high degree of parasitemia in the acute phase of infection is not associated with more severe clinical manifestations of the disease in the chronic phase. However, this high-grade tissue parasitism elicits a potent cellular and humoral immune response against *T. cruzi*, which leads to the immunological control of the parasite, mainly through macrophage and dendritic cell recruitment that promotes phagocytosis of the parasites and expression of interleukin-12 and other proinflammatory molecules [34,35,43]. The parasite persistence induces the recruitment and expansion of *T. cruzi*-specific T cells to the myocardium, particularly Th1, and an increase in cytokines concentration such as interferon-γ [32,34]. Additionally, in vivo murine models show that *T. cruzi*-infected mice display autoantibodies specific for various proteins such as cardiac myosin, desmin, actin, β1-adrenergic, and M2-muscarinic cholinergic receptors, showing the relevance of the humoral response in the development of CD [34,44,45].

## 4. Clinical Manifestations

### 4.1. The Natural History of the Disease

CD is a heterogeneous entity that presents with a variety of clinical manifestations and prognoses. It is characterized by two main phases, acute and chronic. The first signs of infection occur about 1 to 2 weeks after the initial exposure to an infected triatomine bug [46]. When the parasites enter through erosions in the skin, a small number of patients may develop a local inflammatory response that manifests with the development of an indurated area of erythema, swelling, and regional lymphadenopathy known as Chagoma. The Romaña sign is also a typical finding, consisting of unilateral painless periorbital soft tissue edema that occurs when the conjunctiva is the entry portal for the parasite [6].

Local signs may be followed by a non-specific flu-like syndrome characterized by malaise, fever, anorexia, generalized lymphadenopathy, and hepato-splenomegaly. If the transmission occurs via transfusion or transplantation, initial symptoms may appear up to 3 to 4 months after the event [6]. An increased parasite load and advanced age may be associated with these severe presentations [4,6].

The acute or primary infection usually resolves within 8 to 12 weeks after transmission, and it often remains undiagnosed because most patients manifest mild and nonspecific symptoms. Subtle changes may appear on the electrocardiogram (ECG), including sinus tachycardia, prolonged PR and QT intervals, generalized low voltage, and repolarization abnormalities [47]. Circulating trypomastigotes are also detectable, but serum parasite levels fall below the microscopical threshold for detection by the end of the initial phase. Lack of treatment or the immune system’s inability to successfully clear the infection causes patients to enter the chronic phase. This phase is subdivided into four main clinical presentations—indeterminate, digestive, cardiac, or cardio-digestive disease [6].

Up to 70% of infected individuals remain asymptomatic or indeterminate throughout life [48,49]. However, they remain infectious to vectors (serving as reservoirs) and transmit the disease through vertical transmission, blood transfusion, or organ donation. The chronic phase is defined by a positive anti-*T. cruzi* serology in the absence of symptoms or physical signs of the disease, a normal ECG, and no relevant findings during cardiac, esophagic, or colonic imaging [6]. Although patients may persist in this stage for decades, the progression rate to clinically overt disease ranges from 1.5 to 1.9%, with a cumulative progression of 6.9% [50,51]. Some risk factors have been established as triggers for progression and include age, male sex, parasite strain, genetic background, African ancestry, the severity of acute infection, reinfection, nutritional status, alcoholism, and persistence of high parasitemia [6,51].

About 20% to 30% of individuals develop Ch-CMP, 10% to 15% gastrointestinal disease, and a minimum number of patients develop the cardio-digestive phenotype [50,52,53,54]. As mentioned, gastrointestinal involvement depends on the parasite’s geographic-specific circulating genotypes, which explains why it is mainly seen in the countries in the southern region of the Americas [25,26]. The result of impairments on the enteric nervous system causes abnormal esophageal and colonic motility, triggering clinical presentation that ranges from mild achalasia to severe megaesophagus and from mild constipation to megacolon [6,55].

### 4.2. Chagas Cardiomyopathy (Ch-CMP)

Ch-CMP encompasses all patients with CD and cardiac involvement, defined either by typical electrocardiographic abnormalities or signs of dilated cardiomyopathy [6]. Typically, the disease involves all cardiac chambers, with a classical distribution of fibrosis in the basal inferolateral and apical regions of the LV, associated with sinus node and conduction system abnormalities [4,6].

Although there are no pathognomonic electrocardiographic changes for CD, classical ECG findings include a right-bundle branch block (RBBB) with or without a left anterior fascicular block (LAFB). RBBB is one of the first clinical signs that appear and constitutes the most frequent conduction abnormality, seen in up to 50% of patients with chronic disease in the general population, with an odds ratio (OR) of 4.6 [56]. The association of RBBB and LAFB is strongly suggestive of CD with an OR of 3.3 and should motivate an investigation for epidemiological, clinical, and serological factors [56].

These clinical signs usually mark the transition from the indeterminate phase to Ch-CMP, representing an increased risk of disease progression. On the other hand, dilated Ch-CMP is the term used to describe the typical hemodynamic pattern of LV dilation associated with segmental or global systolic dysfunction, regardless of ECG findings [57]. Studies have demonstrated that brain natriuretic peptide (BNP) has a role as a reliable predictor for LV systolic and diastolic dysfunction and is considered the most robust predictor in prospective studies of patients with Ch-CMP [58].

As for clinical manifestations, there is a broad spectrum ranging from asymptomatic presentation to congestive heart failure and arrhythmogenic cardiomyopathy symptoms such as dyspnea on exertion, fatigue, palpitations, dizziness, and syncope [6]. Atypical chest pain that imitates ischemic disease may appear secondary to microcirculation involvement, often accompanied by non-specific ST-segment changes and pathological Q waves in the ECG [56,59,60]. Cardiac examination typically demonstrates murmurs due to functional mitral and or tricuspid regurgitation, wide splitting of the second heart sound, and prominent diffuse apical thrust. Hence, signs and symptoms of Ch-CMP can be categorized into three major clinical syndromes: (1) abnormalities of the electrical conduction generating tachycardia and bradyarrhythmias, (2) myocardial contractile dysfunction resulting in heart failure, and (3) clinical evidence of thrombi formation [4,6].

#### 4.2.1. Tachy and Bradyarrhythmias

Chagas heart disease is characterized by a variety of abnormalities of the conduction system, probably as a consequence of regional fibrosis that shows tropism for the cardiac conduction system (sinus node, AV node, and bundles of His), and the subsequent macro-reentrant circuits that originate in these areas [6,61]. Another possible explanation is the extensive myocardial sympathetic defects, especially along the ventricular myocardium [62]. These changes lead to dilated cardiomyopathy that predisposes the conduction system to electrical abnormalities, which may cause both brady and tachyarrhythmias.

Sick sinus syndrome can manifest as sinus bradycardia, electrical pauses, sinoatrial block, and, in severe cases, tachycardia-bradycardia syndrome. Among supraventricular tachyarrhythmias, atrial fibrillation with a non-rapid ventricular response is the most common. It is found in approximately 10% of patients and is often a marker for advanced myocardial damage, an independent risk factor for stroke, and a strong mortality predictor [47,51,63,64].

However, ventricular tachyarrhythmias are more frequent, affecting up to 65% of individuals [65,66]. Monomorphic and polymorphic ventricular beats, couplets, and non-sustained ventricular tachycardia (VT) are the most frequent findings [67]. Historically, VT has been associated with substantial morbidity and mortality because of their role in SCD, and it was thought that their presence, duration, and complexity were related to the severity of the regional wall motion abnormalities (RWMA). However, in a recent large observational study with more than 100 enrolled patients diagnosed with Ch-CMP, electrical storms (ES), defined as three or more distinct episodes of sustained VT or VF (ventricular fibrillation) within 24 h conferred no difference in mortality [68]. Moreover, a depressed left ventricular ejection fraction (LVEF) was not associated with the presence of ES, and a shorter QRS duration was seen among these patients, probably indicating a more preserved conduction system, which can be a surrogate marker of a less affected LV [68]. Therefore, there is still uncertainty regarding the most prominent factor contributing to ES in Ch-CMP.

Regarding SCD, its impact has always been highlighted in Chagas-endemic populations, almost since its first descriptions. Most studies show that SCD is the most common cause of death among patients with CD, causing up to 55 to 60% of deaths [69]. Sustained VT that triggers and turns into VF is the principal cause of SCD in non-Chagasic cardiomyopathy [70,71]. However, other mechanisms such as the rupturing of apical LV aneurysms, massive pulmonary or brain embolism (due to ventricular aneurysms), and harmful bradyarrhythmias such as advanced AV blocks, sinus node dysfunction, and abnormal Bezold Jarisch reflex activation can also lead to sudden death in Ch-CMP [69,70].

An important histological finding detected in these patients that require further investigations is the higher frequency of myocytolysis, a reaction considered typical of catecholamine toxicity, compared to CD patients who do not experience SCD. This finding may be consistent with the hypothesized role of the autonomic nervous system dysregulation as a mechanism of SCD in Ch-CMP [70].

#### 4.2.2. Ventricular Dysfunction

Heart failure in CD is typically caused by a progressive dilated cardiomyopathy in which RWMA usually precedes global LV dysfunction. The segments most commonly involved are the LV apex and the inferolateral wall [72,73]. Alone, RWMA constitutes a risk for developing ventricular arrhythmias even in the early stages of the disease [73,74]. Right-sided heart failure may also be present, but it is usually due to an increased afterload secondary to LV dysfunction [75,76,77]. When biventricular dysfunction occurs, functional mitral and tricuspid regurgitation may worsen the prognosis [78]. Generally, systolic and diastolic dysfunction coexist in Ch-CMP. Chronic myocarditis first alters ventricular relaxation and diastolic filling and, as the disease progresses, systolic dysfunction appears [79]. Patients usually may manifest with both left and right-sided heart failure, including symptoms such as fatigue, chest pain, dyspnea, pulmonary edema, increased jugular venous pressure, peripheral edema, ascites, and hepatomegaly.

Clinical progression of chronic Chagas heart disease has been classified in four stages, from A to D, based on the severity of the symptoms according to the LATAM guidelines for diagnosing and treating Chagas’ heart disease (Table 2). Patients in the indeterminate form of CD make up stage A, meaning they have risk factors in developing Ch-CMP but are yet to develop heart failure or structural heart disease symptoms. Asymptomatic patients with structural heart disease, defined by either ECG findings or echocardiographic findings, are part of stage B. B1 patients present mild changes with preserved global ventricular function. At the same time, B2 includes patients with decreased LVEF. Once symptoms of heart failure appear, the ventricular function is severely affected. In such cases, patients are reclassified as stage C. Finally, stage D indicates the presence of signs and symptoms that are refractory to medical therapy, therefore, needing specialized and advanced interventions [80].

Ch-CMP carries a poor prognosis, mainly due to the aggressive ventricular remodeling that carries a significant risk for developing arrhythmias and other adverse events. Although the appearance of symptoms is a strong indicator of advanced disease, efforts have been made to develop tools that allow early identification of patients at higher risk of developing complications. Echocardiography is a widely available imaging option that may help establish baseline characteristics regarding all cardiac chambers and LV systolic and diastolic function size. Another high-yield assessment tool is cardiac magnetic resonance (CMR), which has proven to be excellent at imaging cardiac anatomy and function with remarkable ability to determine ventricular size and function and characterize cardiac tissue to identify myocardial fibrosis (MF) [81]. Late gadolinium enhancement (LGE) and T1 sequences provide an excellent depiction of scars, edema, and MF in Ch-CMP [82,83].

MF in Ch-CMP was historically described as having sub-epicardial and mid-wall distributions in the LV. Still, the largest cohorts have demonstrated that it can have any distribution, and the most frequent is transmural, resembling that of myocardial infarction [82,83]. The association of MF and onset of RWMA followed by global dysfunction has been established. Hiss et al. reported the first longitudinal observational study of patients with Ch-CMP showing that the progression of both segmental and global systolic dysfunction correlated with the aggravation of the extent of the perfusion defects and an increase in the areas of MF in the same distribution. The results were posteriorly associated with pathology studies on human hearts showing the MF findings and the degree of cardiac function impairment [74].

Nevertheless, prognosis should not be entirely based on MF since there are other findings that CMR also identifies and are equally important to detect as early as possible on the disease’s natural history, including myocardial edema, aneurysm formation, intracavitary thrombus, and focal and diffuse fibrosis in the noted territories [84,85,86,87].

#### 4.2.3. Chagasic Aneurysms and Thromboembolism

Cardiac aneurysms, most typically located on the LV apex, are seen in up to 8% of asymptomatic cases and 50% of people with moderate to severe myocardial involvement [4,6]. Their presence, disregarding the size, is a significant risk factor for developing mural and intracavitary thrombus and stroke and is a marker for increased mortality [88,89]. Notwithstanding, there is not enough evidence to use them as an independent risk factor for mortality when adjusted for LVEF [90].

Thromboembolic events, particularly to the brain, are relatively frequent among patients diagnosed with CD, and they constitute up to 18% of all stroke cases in Chagas-endemic areas [91]. Its leading cause is thought to be cardioembolic, many of them attributed to the formation of intracardiac thrombi, favored by dilated cardiac chambers and LV aneurysms. Nonetheless, blood flow stasis secondary to an abnormal ventricular function and atrial arrhythmias may also play a significant role. Other established risk factors are left atrial enlargement, older age, and concomitant classical cardiovascular risk factors such as hypertension and dyslipidemia that promote a proinflammatory and prothrombotic state endothelial dysfunction [92,93,94,95,96].

Patients commonly manifest with abrupt onset of anterior circulation syndrome, including motor or sensory focal deficits, homonymous hemianopia, and higher cortical dysfunction such as aphasia or visuospatial deficit. Approximately 30% of patients may present with a posterior circulation syndrome [97]. These events may contribute to further cognitive deterioration and dementia in these patients, besides the one attributed to the infection itself [98,99]. Pulmonary emboli may appear due to venous or right heart thrombi.

## 5. Diagnosis

The diagnosis of CD requires either the detection of the parasite in blood samples or evidence of seroconversion. In the acute phase of infection, serum parasite levels are high, therefore, microscopic examination is the simplest way to identify the trypomastigotes. [100]. The polymerase chain reaction is a sensitive and specific diagnostic in the acute phase of the infection, with a reported sensitivity of 95.7% [101]. Usually, it is possible to obtain positive results even before parasites detect blood smears [102].

Molecular tools are also crucial for diagnosing congenital transmission, oral infections, early detection of infection in receptors of organs from CD donors, monitoring reactivation in immunodeficient patients, and evaluating treatment response [103]. Although blood cultures and xenodiagnosis provide a direct demonstration of the circulating microorganism, they are considered very demanding procedures, and final results may not be available for one or two months. As a result, newer molecular tools have replaced mainly older direct parasite identification methods [100]. Despite these tools’ availability, *T. cruzi* is rarely detected during the acute phase, except in cases of specific screening programs or outbreaks [6].

Levels of parasitemia decrease within 10 to 12 weeks of infection, even without treatment. Therefore, at this stage, molecular tests have a much lower sensitivity at around 64.2% [101]. Hence, serological tests are necessary to diagnose CD in the chronic phase [104]. The definite diagnosis requires two positive laboratory tests with different methods for detecting seroconversion, and if the results are contradictory, a third test is needed to confirm the diagnosis [100]. This process has been established due to the potential of false-positive reactions, typically with samples from patients with other parasitic or autoimmune disorders [100,103]. Some of the available options include indirect immunofluorescence, hemagglutination, and enzyme-linked immunosorbent assay ELISA [100]. The most commonly used technique, ELISA, regardless of the commercial test, has an overall sensitivity and specificity of 97.7% and 96.3%, respectively [105,106]. A next-generation ELISA diagnostic assay based on the combination of short peptidic epitopes rather than parasite lysates, antigenic fractions, or purified recombinant antigens, which usually require high technical knowledge and are expensive to produce, was recently developed by an Argentinian group. It displayed a high diagnostic performance, with a sensitivity of 96.3% and a specificity of 99.1%, and a positive and negative predictive value of 98.7 and 97.4%, respectively [107]. This new approach, compared to the commercially available assay, is an attractive alternative in CD diagnosis.

Regarding screening for underlying heart disease, cardiac function assessment in patients with confirmed that *T. cruzi* infection is essential to detect early cardiac involvement and risk stratification before symptoms develop. Patients should be questioned about the presence of symptoms related to heart rhythm disturbances such as palpitations, dizziness or syncope, heart failure manifestations, systemic thromboembolism, and microvascular disorders such as chest pain. Routine ECG evaluation is essential to identify acute changes, especially those regarding heart rhythm that generally remain asymptomatic for long periods. All patients with an established diagnosis, regardless of their severity, should undergo a 24-h Holter ECG monitoring to assess the presence and frequency of ventricular or supraventricular arrhythmias, sinus node disease, and atrioventricular conduction abnormalities [80,100].

An echocardiogram should also be done, ideally during the indeterminate stage, to establish baseline characteristics for further comparison. It is also favorable to identify early asymptomatic RWMA, the presence of ventricular aneurysms, and to evaluate right ventricular (RV) function [78]. The test should be repeated once patients present with worsening symptoms or new ECG changes. Regarding the presence of thrombi, both transthoracic and transesophageal echocardiograms are frequently required to rule out ventricular and atrial thrombus, particularly in the setting of atrial fibrillation or other supraventricular tachyarrhythmias. Transthoracic images better identify LV aneurysms and thrombi, while transesophageal echocardiograms excel at finding those in the left atria [88]. Other tests such as cardiac stress images may help assess the chronotropic response, which may be affected in the setting of autonomic impairment, and to unmask complex ventricular arrhythmias [108].

CMR is also an excellent option for anatomical and functional evaluation of all cardiac chambers, with several advantages over other imaging modalities, particularly the echocardiogram, because it allows for a more precise measurement of both RV and LV ejection fractions, tissue characterization, and detection of RWMA, thrombi, and aneurysms. However, its accessibility is limited and is only available on very few sites in LATAM. LGE is useful for identifying MF’s distribution and characteristics, which is probably its most important use because of its relation to the severity of the disease. Rochitte et al. described MF findings in a group of 51 patients at different CD stages; 15 patients were considered to be in the indeterminate phase, 26 of them had well-known Ch-CMP, and 10 had Ch-CMP complicated with VT. MF was present in 20% of asymptomatic patients, 84.6% of Ch-CMP, and 100% with Ch-CMP and VT. The progression of MF prevalence across disease severity subgroups suggests its possible role as a severity predictor in Ch-CMP and a higher risk for SCD [93].

More recently, in a study conducted in Sao Paulo with 121 patients, LGE was identified in 78.5% of them. These same patients had a lower LVEF and lower event-free survival. The presence of more than 5% of the scar was an independent predictor of events with a hazard ratio (HR) of 2.2 [83]. The segments that showed LGE was the same that showed RWMA and the ones associated with LV aneurysms in 86% and 67%, respectively [81]. Additionally, the presence of two or more contiguous segments with transmural fibrosis was a VT predictor with a relative risk (RR) of 4.1 [81].

Recent investigations have identified inflammatory and cardiac biomarkers that are differentially expressed in the various clinical forms of CD and may be of clinical value for disease staging. A cross-sectional retrospective case-control study with more than 1000 patients tested 22 biomarkers to identify which ones were associated with Ch-CMP. A clear pattern was found among patients with Ch-CMP, presenting with high levels of inflammatory biomarkers such as IL-10 and IL-6 and markers associated with cardiac dysfunction such as troponin, NT-pro BNP, myoglobin, CK-MB, and adiponectin. Troponin and NT-pro-BNP were significantly increased among patients with CD without established heart disease, suggesting its potential use as early biomarkers of disease progression. NT-pro-BNP alone was the strongest predictor of Ch-CMP, also associated with NYHA functional class, ventricular arrhythmias, and LV dysfunction [58]. Nevertheless, these labs require a high degree of clinical suspicion in order for them to be useful in the clinical setting of CD.

## 6. Risk Stratification and Scores

As a heterogeneous entity, the CD may present with multiple clinical courses, which imply different prognoses. Therefore, prognosis and risk stratification scores should be performed in all CD patients. The Rassi score is a well-known and validated tool that effectively predicts adverse outcomes, specifically long-term risk of death, in patients with Ch-CMP. Its main advantage is that it is solely based on six clinical features, which include: (1) the presence of heart failure symptoms defined as NYHA class III or IV, (2) cardiomegaly evidenced on chest radiography, (3) LV systolic dysfunction evidenced as segmental or global RWMA on echocardiogram, (4) non-sustained VT, (5) low QRS voltage, and (6) gender. The scores derived from the presence of these risk factors help to classify the patients into subgroups of low (0 to 6 points), intermediate (7 to 11 points), and high risk (12 to points) for death with 10-year mortality rates of 10%, 44%, and 84%, respectively [109] (Table 3).

Senra et al. more recently validated the Rassi score with high performance in patients with Ch-CMP by comparing it with the prognostic value of MF when measured by CMR for predicting all-cause mortality and combined challenging events in over 100 patients within five years. The study concluded a rate of all-cause mortality of 11%, 33%, and 57% among the low, medium, and high-risk groups as stratified by Rassi score, respectively, which somehow matched the previous results found in 2006 and defined it as a stronger predictor of hard events than MF severity and complexity. Another relevant finding was the score’s ability to independently predict risk for heart transplantation, anti-tachycardia pacing, or appropriate shock from an implantable cardioverter-defibrillator (ICD) and aborted SCD [110].

As previously mentioned, different findings support the use of CMR as a non-invasive alternative for stratifying the risk of adverse outcomes in patients with Ch-CMP. Its utility has been demonstrated beyond its correlation with the Rassi score in multiple studies, suggesting its cost-effectiveness and encouraging its systematic use in high-risk patients. Its major problem is, as mentioned, its limited availability in low-income areas throughout LATAM. In the meantime, the duration of the QRS interval has also been directly correlated with the scar and MF size estimated by LGE. Therefore, ECG can also be presented as an attractive screening tool and may allow a simple, first-step process for these patients’ risk stratification [111].

More recently, the Sousa score has been proposed to predict SCD in patients with chronic Chagas heart disease. It includes four independent factors: (1) the QT-interval dispersion defined as the difference between the maximum and minimum QT interval (three points); (2) presence of syncope (two points); (3) ventricular extrasystoles (one point); (4) and severe LV dysfunction (one point). Patients are then classified as low (zero to two points), intermediate (three to four points), or high risk (greater than five points). The rates of SCD in these groups after a mean 5.5-year follow-up were established at 1.5%, 25%, and 51%, respectively. The score is also useful for treatment guidance due to the potential benefit of advanced therapies, including an electrophysiological study among high-risk patients [94] (Table 2).

Finally, the IPEC/FIOCRUZ score was developed in 2008 to identify patients with Ch-CMP at high risk of stroke and initiate prevention strategies when needed. Four items were identified as predictors of stroke: (1) systolic dysfunction (two points), (2) age > 48 years, (3) primary alteration of ventricular repolarization, and (4) apical aneurysm of the LV (one point each). The score ranges from zero to five points, and the study suggested primary prophylaxis with warfarin (INR 2-3) for those scoring four or more and aspirin or warfarin for those with three points (moderate risk) [95]. However, a revalidation study conducted in 2016 suggested that the score might be underestimating the risk of thromboembolic events and classifying inappropriately high-risk patients in moderate or even low-risk groups, raising concern for its widespread use [96] (Table 2).

## 7. Current Treatment and Prognosis

### 7.1. Antimicrobial Therapy

Nowadays, the only two anti-trypanosomal regimens available with proven efficacy against CD are benznidazole and nifurtimox. Both medications are contraindicated during pregnancy, which forces treatment to be delayed until delivery [112]. Ongoing trials such as the EQUITY trial will inform the trypanocidal effect and equivalence of both of these drugs, establishing which one should be used as a first-line treatment [113].

As of today, benznidazole is usually considered the first-line therapy because it has significantly more evidence supporting its efficacy for parasitic clearance, is generally better tolerated, and is widely available [114,115]. A dose of 5 mg/kg per day for 60 days is recommended for chronic cases and 10 mg/kg for acute patients. Only about 22% of excretion is fecal. There are no hepatic or renal dosage adjustments [6].

Although generally well-tolerated, side effects of benznidazole include mild gastrointestinal disturbances such as nausea or vomiting that can lead to significant weight loss, skin hypersensitivity manifested as dermatitis, leukopenia, self-limited peripheral neuropathy, which often appears near the end of the regimen, anorexia, and insomnia. A complete blood count should be performed approximately 21 days after treatment initiation to monitor the possibility of leukopenia. In most cases, dermatitis can be controlled with prednisone 10 mg once daily for ten days. However, treatment should be discontinued in severe cases. Concurrent alcohol use can lead to a disulfiram-like effect, and its consumption should be avoided in all cases [6,116,117].

Nifurtimox is the other available drug against *T. cruzi* infection and should be kept as a second-line treatment in cases of benznidazole toxicity. Its dose ranges from 8 to 10 mg/kg daily in 3 to 4 oral doses for 60 days. In children <11 years old, the recommended dose is 15–20 mg/kg per day, and from ages 11–16, an amount of 12.5 to 15 mg/kg. Its excretion is mainly renal (from 27% to 44%, depending on fasting/fed conditions), primarily as metabolites. Although its concentrations may be increased in patients with end-stage renal disease on dialysis and should be used with caution, there are no renal dosage adjustments. Its most common side effects, as with benznidazole, are gastrointestinal complaints such as anorexia, nausea, vomiting, abdominal pain, diarrhea, and weight loss. Central nervous system toxicity is also common, manifesting with irritability, insomnia, disorientation, drowsiness, and psychiatric disorders. Less common side effects include peripheral neuropathy, paresthesia, and tremors, which are dose-dependent and often occur during the second month of treatment, requiring therapy discontinuation or dose adjustment. Although disulfiram-like effects are not present, the drug is metabolized by the cytochrome P450 system, which increases the possibility of severe pharmacological interactions [6,117,118].

Anti-trypanosomal treatment is indicated in all patients with acute CD as soon as the diagnosis is made. Parasite clearance has been estimated between 60 and 100%. However, treatment rarely leads to complete *T. cruzi* eradication [6,119]. In severe acute presentations, the treatment should be accompanied by hemodynamic support.

On the other hand, the role of anti-trypanosomal treatment in chronic CD and Ch-CMP is highly controversial. Current guidelines recommend treatment in the early, undetermined stages. However, cure rates in this phase are not as good, and the goal is to prevent the development of chronic manifestations by reducing the parasite burden. The BENEFIT trial was a multinational, multicenter, randomized controlled trial, which enrolled 2854 patients and was conducted from 2004 to 2011 in five different LATAM countries. It aimed to evaluate the efficacy and safety of benznidazole as compared with a placebo among patients with Ch-CMP. This study showed that despite reductions in the serum parasite load on the treatment group, with rates of 66% at the end of treatment and 46.7% after five years or more, there were no statistically significant differences between treatment and placebo groups in outcomes of death, aborted SCD, sustained VT, insertion of a pacemaker or implantable cardioverter-defibrillator, cardiac transplantation, new-onset heart failure, stroke, or other thromboembolic events. In light of this, anti-trypanosomal therapy is generally not prescribed to patients with advanced cardiomyopathy as any clinical benefit is likely negligible, and there is a significant risk of adverse effects. Exceptions for this recommendation include women of childbearing age to prevent congenital transmission and immunocompromised patients with reactivation of the disease [120].

Currently available therapies are far from ideal despite appropriate adherence, especially because of their side effects, which can interrupt the therapeutic protocol and affect the treatment’s compliance, and its limited efficacy on parasitic clearance. Other factors that influence efficacy include treatment regimen, patient’s age and immune system and geographical origin, which may increase the risk of infection with a certain T. cruzi strain with natural resistance to both drugs [119]. Treatment may also be limited by the social determinants of health such as poverty and social vulnerability, which, as of today, have not been extensively studied nor effectively intervened [121].

Limitations of the current mainstay medications highlight the fact that more research is needed to discover both new drug targets in *T. cruzi* and new drugs against Chagas disease. Progress in developing and testing drug candidates, as well as studies adapted to LATAM’s socio-economic context should be encouraged, in order to shed light on true efficacy and adherence. Since the introduction of these drugs, only allopurinol and the azoles itraconazole, fluconazole, ketoconazole, posaconazole, and ravuconazole have been studied in clinical trials, observational studies, or clinical cases, with inconclusive and disappointing results in phase II clinical trials. An alternative approach is to study the modification of the current therapies in order to diminish their toxicity as well as to increase their trypanocidal efficacy, and/or their combination of the most promising azoles (posaconazole and ravuconazole) [119].

Strategies involving gene editing by the CRISPR/Cas9 nuclease system are being studied because of the potential ability to generate modified *T. cruzi* cell lines, with knockout, complementation, and in situ tagging of *T. cruzi* genes involved in the parasite’s life cycle [122,123]. As of today, these methods have been described in the generation of mutant cell lines with genome editing of proteins involved in calcium homeostasis [123]. However, it is possible to assume that in the future, we may be able to obtain non-infective trypomastigotes and intracellular amastigotes.

### 7.2. Neurohormonal Blockade and Heart Failure Treatment for Ch-CMP

Most medical treatment for patients with Ch-CMP has been extrapolated from data on other heart failure forms, especially non-ischemic causes of dilated cardiomyopathy, because of the lack of formal trials regarding whether renin-angiotensin system (RAS) inhibitors and β-blockers are safe and beneficial in Ch-CMP. Although there is common pathophysiology that suggests that treatments shown to be effective in other forms of cardiomyopathy should also be helpful in Ch-CMP, several characteristics such as early cardiac parasympathetic denervation, cardiac-muscle hypertrophy, and dilation, and severe focal fibrosis, may lead to the perception that usual drugs could be contraindicated. The first randomized trial regarding this topic was conducted in 2007, with 42 patients, in which investigators first analyzed the effects of treatment with enalapril and spironolactone and then undertook a randomized trial of adding carvedilol. It was demonstrated that the combination of medications was safe, hemodynamically and clinically well-tolerated, and associated with improvements in cardiac function and clinical status [124]. Other efforts have been made to assess CD patients’ response to digitalis, diuretics, vasodilators, and β-blockers; none of them have had enough quality to issue strong recommendations. In a post hoc analysis of the PARADIGM-HF trial (comparing sacubitril/valsartan against enalapril in patients with reduced LVEF heart failure), a total of 113 patients with Ch-CMP were randomized, 58 to the sacubitril/valsartan arm and 55 to enalapril. Sacubitril/valsartan was associated with a reduced risk of cardiovascular death or heart failure hospitalization compared to enalapril. Noteworthy, this trial was underpowered to establish a strong recommendation for this treatment [125]. Notwithstanding, it provided some insights regarding the potential benefits which needed to be proved in more specific trials. The PARACHUTE—HF trial, a multicenter, prospective, phase 4 study, was conducted to evaluate the effect of sacubitril/valsartan compared to enalapril, in addition to conventional heart failure medication, in improving the rate of cardiovascular events and reducing levels of NT-proBNP, in Ch-CMP participants with a reduced ejection fraction [126]

As of today, treatment is based on a standard neurohumoral blockade with angiotensin-converting enzyme inhibitors (ACE-I) or angiotensin II receptor blockers (ARB), β-blockers, and mineralocorticoid receptor antagonists for patients with NYHA III or IV [127]. Specifically, β-blockers play a crucial role in this disease for all symptomatic or previously symptomatic patients with reduced LVEF because of their role in adrenergic response modulation [124]. Unfortunately, many patients with Ch-CMP have low baseline heart rates or use amiodarone because of ventricular arrhythmias and may not tolerate guideline-directed dose titration of β-blockers. However, it should be noted that evidence suggests titrating β-blockers up to their maximum dose before initiating any antiarrhythmic medication [124].

Regarding VT/VF, the best approach to reduce the incidence of life-threatening arrhythmias and SCD is ICD implantation plus amiodarone, especially for secondary prevention, after documented VT, VF, or aborted SCD [127,128]. Together, both therapies have improved outcomes, as shown by Gali et al. in 2013, who demonstrated that adjunctive treatment with a defibrillator reduced the risk of all-cause mortality by 72% and the risk of SCD by 95%, compared with amiodarone-only therapy. Both approaches have been empirically and commonly used for primary prevention with mixed results, especially in patients with LVEF lower than 40% [128]. Currently, the ATTACH trial is ongoing to evaluate whether treatment with amiodarone has a trypanocidal effect and exerts a clinical benefit in terms of mortality and incidence of arrhythmic cardiac event, among individuals with mild-to-moderate Ch-CMP [129]. Regarding ICD therapy on its own, in a 2019 meta-analysis conducted by Rassi et al., which included only observational studies, ICD implantation was found to be associated with an annual all-cause mortality rate of 9%. Additionally, appropriate interventions (either shocks or ATP) had a rate of 25% per year. However, inappropriate shocks were not infrequent (5% per year) [130]. The CHAGASICS trial, a study designed to evaluate the benefit of ICD in patients with advanced Ch-CMP, will soon begin recruitment; thus, although promising, the evidence is yet scarce regarding the role of ICD in patients with cardiac involvement due to Chagas disease [131]. Additionally, ablation of VT, either surgical or catheter-based, is also an option to treat recurrent and refractory VT despite antiarrhythmic drug therapy or if these drugs are not tolerated or undesired [127].

Symptomatic sick sinus syndrome or advanced AV blocks are indications for pacemaker implantation. Resynchronization therapy should also be performed according to general heart failure guidelines, especially when patients present with the left bundle-branch block (LBBB) [6,127]. Anticoagulant therapy for stroke prophylaxis is indicated in patients with concomitant atrial fibrillation with CHA2DS2-VASc score ≥2. Additional recommendations have been issued, and they include the presence of thrombi, stroke, or ischemic attack, especially in the presence of an aneurysm. The IPEC/FIOCRUZ score may guide the decision. However, it is highly controversial and should be individualized considering the patient’s risk of hemorrhage and patient’s preferences [6,95].

### 7.3. Advanced Therapies in Ch-CMP

Ch-CMP is associated with lower survival rates than other forms of cardiomyopathy of similar severity. Advanced options, such as ventricular assistance devices and heart transplantation (HT), should be considered for patients whose medical therapy has failed.

Assistant devices such as left ventricular assist devices (LVAD) are an option for patients with end-stage Ch-CMP, either as a bridge to transplant or as destination therapy. Notwithstanding, published data is scarce, and experience is minimal. Therefore, there is no consensus regarding the best strategy. Moreira et al. were the first to suggest the role of LVAD as a valuable treatment option for patients with Ch-CMP who evolve with decompensated heart failure or cardiogenic shock. In this case, they included six patients and obtained contrasting results [132]. Experience with a total artificial heart for more than six months has also been reported more recently by Ruzza et al., followed by successful orthotopic HT [133].

Transplantation is the other option; however, it represents a challenge in patients with CD due to the risk of *T. cruzi* reactivation in the context of immunosuppression. As of today, there is a lack of consensus regarding the diagnosis of reactivation episodes in transplanted patients (Table 4). The diagnosis can be made when symptoms suggesting CD appear (in the form of myocarditis, panniculitis, meningoencephalitis, new skin nodules, or even acute-like symptoms such as fever or jaundice), along with positive detection of parasites in blood or cerebrospinal fluid through PCR analysis, or tissue biopsy, including endomyocardial biopsy (EMB). An increase in parasitemia, detected either by direct parasitological techniques or by PCR, should be documented. PCR technique shows sufficient sensitivity (82%) to detect a reactivation before complications develop [102]. Diagnosis can be established when a recent PCR is positive, and the previous result was negative, or if the former test showed lower parasitemia than the current one. However, as patients can show positive PCR in blood, probably due to parasite persistence and fortuitous blood circulation, the one proposed criterion for the diagnosis of reactivation is sequential positive blood PCR results (at least two) of increasing parasitic load in EMB. The cut-off value for reactivation has been considered positive for *T. cruzi* DNA when the parasitic load was >2.00 × 10^−3^ copies per reaction [134]. In some specialized centers, it has been suggested that an increase of more than two standard deviations in quantitative PCR correlates with an increment in the parasite load, therefore, suggesting a reactivation process. However, as of today, this practice is based on anecdotal evidence and has not yet been proved. In asymptomatic cases, risk factors should be considered, such as a previous rejection episode, the presence of malignancy, and any other immunosuppressive condition [135]. The effectiveness of prophylactic therapy with benznidazole before HT was studied in a cohort of 53 patients in Brazil, which were followed for a period of 18 years. Of these patients, 18 received prophylactic therapy and only two of them (11.1%) were diagnosed with CD reactivation. However, among the group without prophylaxis, 45.7% of them (16 patients) were diagnosed with CD reactivation. Therefore, these findings suggest that the use of prophylactic therapy before HT could reduce the incidence of reactivation (OR = 0.12) [136]. However, further controlled randomized trials including multiple centers should be conducted to further study this possible therapy and its more adequate regimen.

Once reactivations are diagnosed, they can be easily treated with benznidazole to prevent the development of infection and posttransplant morbidities. These facts probably explain why, despite the high risk of reactivation, these patients seem to have better survival chances compared with patients with other forms of cardiomyopathy. Survival rates are up to 83%, 71%, 57%, and 46%, at the 1 month, 1 year, 4 years, and 10 years follow-ups [141,142].

Despite the satisfactory results of HT obtained in the treatment of Ch-CMP, candidacy for surgery and organ availability in LATAM pose a significant delay for this treatment. Although there is evidence of accidental cases of unknown donors positive for CD [143,144,145,146,147], it has been documented that the risk of CD transmission is in the range of 15 to 22%, with a good outcome with early diagnosis and treatment [147,148,149,150]. Given the scarcity of organs, some transplant programs and guidelines support the use of kidneys and livers from chronically infected donors, yet they reject intestines and hearts given a 75% chance of high transmission of CD [151,152]. In these cases, prophylaxis is controversial, with limited data in the literature, and it is not recommended [153,154]. To monitor the transplant recipient with potential CD, a laboratory follow-up (parasitemia) is performed weekly until two consecutive negative results are obtained subsequently. It is also requested to perform periodic monitoring for life and post-treatment, which includes serology to detect negative seroconversion that can last from months to years, or never arrives. The cure rate for acute CD/reactivation in these cases is 80% [80,155,156].

In selected patients, particularly those with severe pulmonary hypertension due to severe functional mitral regurgitation, a Mitral Clip can be used as a bridge option to decrease the severity of mitral valve dysfunction and ultimately pulmonary pressure to permit enlisting patients for HT [157].

Treatment for Ch-CMP, especially in its advanced stages, is complex. Further research is needed regarding this topic. However, today, bridging strategies seem reasonable, primarily because of their role in reducing pulmonary vascular resistance to make patients eligible for HT [158].

## Figures and Tables

**Table 1:** Estimated epidemiological parameters of CD in different countries by 2010.

Countries	Estimated Prevalence	Estimated Number of Infected Individuals	Estimated Number of People with Ch-CMP	Estimated Population at Risk of *T. cruzi* Infection
Bolivia [8]	6.10%	607,000	121,000	586,000
Argentina [8]	3.61%	1,505,000	376,000	2,243,000
Paraguay [8]	2.1%	185,000	33,000	1,704,000
Ecuador [8]	1.38%	200,000	40,000	4,200,000
Colombia [8]	0.95%	438,000	131,000	4,814,000
Mexico [8]	0.78%	876,000	70,000	23,475,000
Brazil [8]	0.61%	1,157,000	231,000	25,474,000
USA [11,12]	0.097% *	238,000–300,000	30,000–45,000	NDA
Europe [13,14]	0.01–4.2%	98,000	975,000–54,000	NDA

NDA: No data available, * This parameter was calculated based on population data from 2010. This information is an estimate based on the percentage of people with CD that are likely to develop Ch-CMP.

**Table 2:** Stages of Ch-CMP.

A (Indeterminate Form)	B (Asymptomatic Patients with Structural Cardiomyopathy Defined by Either ECG Findings or Echocardiographic Findings)		C	D
Patients at risk of developing Ch-CMP (established diagnosis) but without symptoms of heart failure or gastrointestinal disease, no structural heart disease, and a normal electrocardiogram	B1	B2	Patients with symptoms of heart failure because of a highly affected ventricular function	Patients with refractory symptoms of heart failure and therefore need specialized and advanced interventions
	Patients with mild changes but with preserved global ventricular function	Includes patients with decreased LVEF		

**Table 3:** Scores useful for evaluating patients with Ch-CMP.

	Rassi Score	Sousa Score	IPEC/FIOCRUZ Score
Utility	Predicts adverse outcomes, specifically long-term risk of death	Predicts SCD and helps define proper initiation of advanced therapies, including an electrophysiological study	Identifies patients at high risk of stroke and defines initiation of prevention strategies when needed
Criteria	- NYHA III/IV (5 points) - Cardiomegaly (5 points) - LV systolic dysfunction (3 points) - Non-sustained VT on 24-h Holter monitoring (3 points) - Low QRS voltage on ECG (2 points) - Male sex (2 points)	- QT-interval disper-sion (3 points) - Presence of syncope (2 points) - Ventricular extra-systoles (1 point) - Severe LV dysfunc-tion (1 point)	- Systolic dysfunction (2 points) - Age > 48 years (1 point) - Primary alteration of ventricular repolarization (1 point) - Apical aneurysm of the LV (1 point)

**Table 4:** Chagas disease reactivation surveillance protocols after solid organ transplantation.

Chagas Disease Reactivation Surveillance Protocol after Solid Organ Transplantation			
Country	Year Published	Tests	Periodicity
Argentina [137]	2012	qPCR Strout method Blood specimen microscopy	Pre-transplant Weekly for 3 months Monthly for the 1st year Biyearly thereafter
Brazil [138]	2015	qPCR Blood specimen microscopy Chagas Antibodies Xenodiagnosis	Pre-transplant Every 3 months for the 1st year Every 6 months thereafter
Spain [139]	2011	qPCR Strout method Chagas Antibodies	Pre-transplant Weekly for 2 months Bimonthly until the 6th month Yearly after the 6th month
United States (CDC) [140]	2011	qPCR Blood specimen microscopy	Pre-transplant Weekly for 2 months Biweekly in the 3rd month Monthly until (at least) the 6th month

It should be noted that there currently does not exist a consensus regarding the definition of Chagas disease reactivation in patients who have received a solid organ transplant.
